# 2484. Clinical and genomic comparison of nosocomial-onset and community-onset listeriosis: a nationwide retrospective study in Japan, 2011-2021

**DOI:** 10.1093/ofid/ofad500.2102

**Published:** 2023-11-27

**Authors:** Koh Shinohara, Yusuke Tsuda, Soichi Arasawa, Yasuhiro Tsuchido, Satomi Yukawa, Taro Noguchi, Masaki Yamamoto, Yasufumi Matsumara, Miki Nagao

**Affiliations:** Kyoto University Graduate School of Medicine, Kyoto, Kyoto, Japan; Kyoto University Graduate School of Medicine, Kyoto, Kyoto, Japan; Kyoto University Graduate School of Medicine, Kyoto, Kyoto, Japan; Kyoto University Graduate School of Medicine, Kyoto, Kyoto, Japan; Kyoto University Graduate School of Medicine, Kyoto, Kyoto, Japan; Kyoto University Graduate School of Medicine, Kyoto, Kyoto, Japan; Kyoto University Graduate School of Medicine, Kyoto, Kyoto, Japan; Kyoto University Graduate School of Medicine, Kyoto, Kyoto, Japan; Kyoto University Graduate School of Medicine, Kyoto, Kyoto, Japan

## Abstract

**Background:**

Sporadic, non-clustered, nosocomial-onset (NO) listeriosis has emerged in immunocompromised hosts. However, most previous reports were single-centered and nationwide study is lacked. The genomic investigation of *Listeria monocytogenes* isolates from NO and community-onset (CO) cases is also scarce.

**Methods:**

We conducted a nationwide retrospective study of consecutive listeriosis cases from 2011 to 2021, in which 24 hospitals participated. Clinical characteristics and outcomes were reviewed. The preserved *L. monocytogenes* isolates were investigated using whole genome sequencing. NO was defined as onset of listeriosis symptoms after third day of admission for medical conditions other than listeriosis. Fisher’s exact test and Mann-Whitney U test were used to compare categorical and continuous variables, respectively.

**Results:**

A total of 195 cases were included. All 11 perinatal cases were NO. Among 184 non-perinatal cases, 31 cases (17%) were NO cases. Clinical characteristics of the NO and CO cases were summarized in Table. In NO cases, median age was 73 years and male patients accounted for 55%, which were similar to those in CO cases. Median days of admission to onset was 10 (range, 3-127). NO cases significantly associated with immunosuppressive medical conditions, especially patients with solid organ tumor and recipients of cytotoxic chemotherapy. NO cases were less likely to involve central nervous system (CNS). In-hospital mortality was similar among both groups. A total of 95 isolates (NO cases: 19 isolates, CO cases: 76 isolates) were included in genomic analysis. A pair of isolates were suspected as clustered cases. 14 clonal complexes (CC) were identified in NO isolates and 23 CCs were in CO isolates (Figure). CC1, the most prevalent hypervirulent clone, was less likely to be found in NO cases; 1 isolate (5%) in NO cases and 20 isolates (26%) in CO cases.

**Figure:**
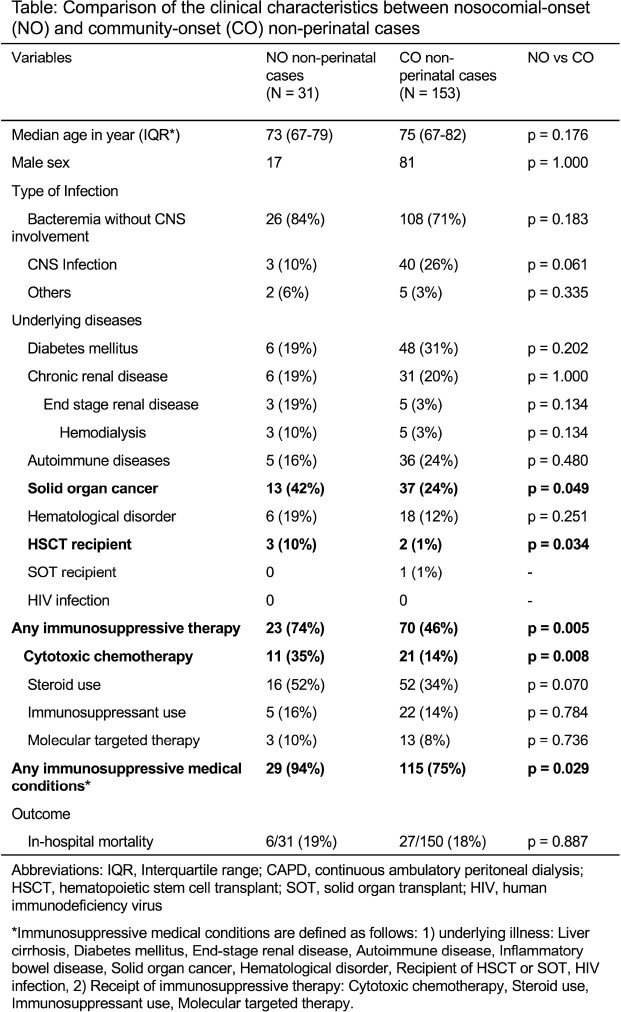

**Figure:**
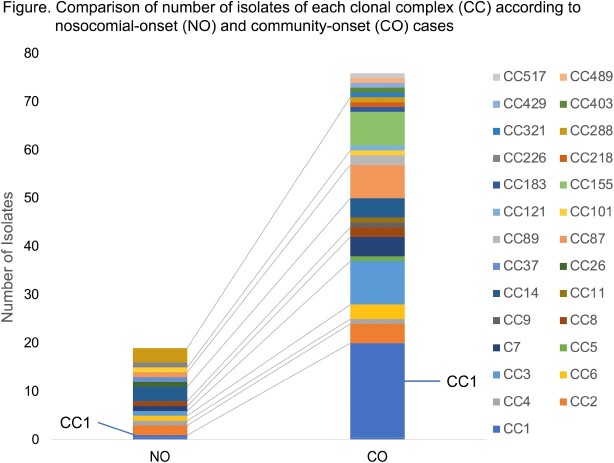

**Conclusion:**

In a retrospective nationwide surveillance in Japan, 17% of listeriosis was NO and mostly affected patients with immunosuppressive medical conditions. Genomic analysis revealed that NO isolates were less likely to be CC1 and have wider genomic diversity than that in CO isolates.

**Disclosures:**

**Yasufumi Matsumara, MD, PhD**, Beckman Coulter: Grant/Research Support|Presicion System Science: Grant/Research Support|Toyobo: Grant/Research Support

